# The impact of CK-MB elevation in patients with acute type A aortic dissection with coronary artery involvement

**DOI:** 10.1186/s13019-022-01924-5

**Published:** 2022-07-06

**Authors:** Naoshi Minamidate, Noriyuki Takashima, Tomoaki Suzuki

**Affiliations:** Department of Cardiovascular Surgery, Shiga University of Medical Science, Setatsukinowa, Otsu, Shiga 520-2192 Japan

**Keywords:** Acute coronary artery involvement, Coronary malperfusion, Acute type A aortic dissection, Coronary artery bypass grafting

## Abstract

**Background:**

Acute type A aortic dissection (ATAAD) is a fatal disease and requires emergency surgery. In particular, it is known that mortality is high when a coronary artery is involved. However, the degree of myocardial damage of the coronary acute artery involvement (ACI) varies and may or may not increase creatine kinase muscle and brain isoenzyme (CK-MB). It is unknown how CK-MB elevation affects the surgical outcome. This study compared the surgical results between the two groups of ACI with or without CK-MB elevation.

**Methods:**

Among 348 patients who underwent an emergency operation for acute type A aortic dissection, there were 28 (8.0%) patients complicated by ACI and underwent additional coronary artery bypass grafting. We divided 26 of those patients into two groups; the MI group ( with CK-MB elevation) and the NMI group (without CK-MB elevation), and compared both groups.

**Results:**

Of the 26, sixteen were in the MI group, and ten were in the NMI group. The average CK-MB in the MI group was 225.5 IU/L, and that in the NMI group was 13.5 IU/L. The mean time from onset to surgery was 248 min in the MI group and 250 min in the NMI group. There was statistical significance in mortality ( 69% vs. 13%, *p* = 0.03). There was no significance in major complications (ICU days, reintubation, reoperation, pneumonia, sepsis).

**Conclusions:**

Acute coronary artery involvement was associated with 8.0% of patients with ATAAD, and 62% had myocardial ischemia with CK-MB elevation. The MI group had significantly higher mortality than the NMI group. It is crucial for cases with suspected ACI to obtain coronary perfusion as soon as possible to prevent CK-MB from elevating.

## Introduction

Acute type A aortic dissection (ATAAD) is lethal and requires emergency intervention. Although recently, the treatment of ATAAD has provided better outcomes than before, acute coronary artery involvement (ACI) is an independent predictor of mortality [1].

Although there were many articles about ACI, the definition of ACI differs because there were no criteria for diagnosis. It was reported that ACI did not necessarily cause myocardial ischemia because the degree of impairment varied from case to case. There was no paper focusing on the actual impact of myocardial damage on patients with ACI. Therefore, in this study, we retrospectively analyzed the surgical outcomes of ACI with or without myocardial damage.

## Material and methods

### Patient population

Between October 2007 and August 2018, 348 consecutive patients underwent surgery for ATAAD in our institution. Twenty-eight patients suffered from ACI and underwent aortic surgery and additional coronary artery bypass grafting (CABG). Patients treated by repair of the coronary orifice (*n* = 4) and those treated by the percutaneous coronary intervention (*n* = 3) were excluded because the surgical treatment background was compared under the same conditions.

### Diagnosis

The diagnosis of ATAAD was confirmed with computed tomography in all cases. The diagnosis of ACI was defined as follows:

- 1. New ST change in the electrocardiogram
- 2. Intraoperative abnormal wall motion of ventricle ( described in the operative record)
- 3. Direct observation of dissected coronary artery after circulatory arrest

We did not perform preoperative coronary angiography routinely, but it was performed in other hospitals for suspected acute coronary syndrome cases. In those cases, ATAAD was diagnosed during the percutaneous coronary intervention (PCI) procedure.

We analyzed laboratory data immediately before surgery and immediately after surgery. According to the results, we divided the patients into two groups:

MI group: with CK-MB > 22 IU/L.

NMI group: with CK-MB < 22 IU/L.

CK-MB over 22 IU/L was the cutoff value in our facility.

### Patient transportation and preparation

Our institute accepted any patients in any condition. Exclusion criteria for operation included patient’s refusal and long cardiac arrest time (> 20 min) before hospital arrival. Patients diagnosed with ATAAD at our hospital were transferred to the operation room directly from computed tomography examination, and patients diagnosed before arrival were taken directly to surgery.

The operative procedure started with median sternotomy and standard extracorporeal circulation (3). Main arterial cannulation sites were via a femoral artery, axillary artery, or the ascending aorta. The femoral vein and right atrium were used for venous drainage. Retrograde cardioplegia was performed via the coronary sinus. The circulatory arrest was achieved at a tympanic temperature of 25–27_C. Then the ascending aorta was incised, and the first retrograde cardioplegia was injected. Basically, we performed ascending aorta replacement. We did not use selective cerebral perfusion to shorten the operative time. After the circulatory arrest, distal anastomosis was made with 4–0 monofilament continuous sutures. Teflon (DuPont, Wilmington, DE) felt strip was used to reinforce the anastomosis of graft and native aorta. Then antegrade systemic circulation was started via the side branch of the graft, and the patient was rewarmed. Finally, proximal anastomosis was performed, and then coronary circulation started after de-airing. CABG was added after aortic repair or before coronary circulation restarted. Intra-aortic balloon pumping necessary was inserted after cardiopulmonary bypass weaning if necessary. It was not used for patients with a patent false lumen of descending aorta.

### Statistical analysis

We analyzed all data with SPSS software version 22.0 (IBM Corp., Armonk, NY, USA). Descriptive data are presented as counts (percentages), means, and standard deviations for normally distributed data, or medians (25th-75th quartile) for skewed data. The primary endpoint was in-hospital mortality, and the secondary endpoint was major complications (reintubation, reoperation for bleeding, postoperative renal insufficiency, pneumonia, and sepsis).

## Results

Baseline patient characteristics are shown in Table 1. The average CK-MB was 226.5 IU/L in the MI group and 13.5 IU/L in the NMI group. No significant difference was observed in most, but only heart failure was significantly more in the MI group.

**Table 1:** Preoperative patient characteristics

Variable	MI		NMI		*p* value
	16		10		
Age	62	± 15	69	± 10	0.63
Male	12	75.0%	6	60.0%	0.42
Height	166.7	± 6.8	160.1	± 13.2	0.22
Weight	66.8	± 11.8	62.1	± 16.5	0.61
Body mass index	23.9	± 3.2	23.7	± 4.2	0.71
Body surface area	1.7	± 0.2	1.6	± 0.3	0.56
ECG ST change	11	84.6%	5	50.0%	0.09
Hypertension	11	68.8%	7	70.0%	0.94
Smoking	6	37.5%	4	40.0%	0.89
CardioHistory	1	6.3%	0	0.0%	0.42
Diabetes mellitus	2	12.5%	1	10.0%	0.72
Hyperlipemia	1	6.3%	4	40.0%	0.03
Preoperative renal insufficiency	0	0.0%	2	20.0%	0.06
Shock	8	50.0%	1	10.0%	0.03
Resuscitation	2	12.5%	1	10.0%	0.72
Heart failure	10	62.5%	1	10.0%	0.01
Arrhythimia	2	12.5%	1	10.0%	0.72

*ECG* Electrocardiogram

Operative data are shown in Table 2. There was no significant difference in the involved artery between the right coronary artery and the left coronary artery. All patients with simultaneous right and left coronary artery involvement were observed in the MI group.

**Table 2:** Operative data

	MI		NMI		*p* value
Ascending replacement + CABG					
RCA	7	43.8%	8	80.0%	0.07
LCA	5	31.3%	2	25.0%	0.52
RCA, LCA	4	25.0%	0	0.0%	0.09
IABP	7	43.8%	0	0.0%	0.01
Percutaneous crdiopulmonary support	2	12.5%	0	0.0%	0.24
Operative time (minutes)	209	± 84	207	± 40	0.46
Circulatory arrest time	25	± 5	25	± 4	0.47
Cardiopulmonary bypass time	152	± 27	150	± 28	0.43
Onset to Operation	248	151–355	250	223–304	0.38
Other artery's involvement					
Cerebral	8	50.0%	3	30.0%	0.31
Mesenteric	3	18.8%	0	0.0%	0.14
Renal	3	18.8%	0	0.0%	0.14
Lower extremities	2	12.5%	0	0.0%	0.24

Total operative time was 209 ± 84 min in the MI group and 207 ± 40 min in the NMI group. Circulatory arrest time was 25 ± 5 vs. 25 ± 4 min, and the cardiopulmonary bypass time was 152 ± 27 vs. 150 ± 28 min, respectively.

In all cases, ascending aorta replacement was performed. Intra-aortic balloon pumping was used in 7 (43.8%) in the MI group. Percutaneous cardiopulmonary support was used in two cases in the MI group. Intra-aortic balloon pumping and percutaneous cardiopulmonary support were not used for patients in the NMI group. Sixteen (11 in the MI group, 5 in the NMI group) patients had other vessels' involvement. Cerebral artery involvement was the most; 8 (50.0%) vs. 3 (30.0%) (*p* = 0.31) respectively. Three patients were in a coma preoperatively. Artery involvement of mesenteric, renal, and lower extremities areas was seen only in the MI group. No intervention for the other artery's involvement was performed.

Table 3 shows the mortality and major complications. The mortality was significantly higher in the MI group than in the NMI group ( 69% vs. 13% *p* = 0.03). The mean ICU stay days were 4 ± 5 days in the MI group and 3 ± 3 days in the NMI group. There was no significant difference in other complications.

**Table 3:** Postoperative data

	MI		NMI		
Postoperative max CK	2956		604.5		
Postoperative max CK-MB (average)	226.5		13.5		
ICU stay (days)	4	± 5	3	± 3	0.41
Total ventilator time (hours)^*^	57	(1–689)	35	(22–440)	0.32
Reintubation	1	(6%)	1	(10%)	0.72
Reoperation for bleeding	0	(0%)	0	(0%)	NS
Postoperative renal insufficiency	0	(0%)	0	(0%)	NS
Pneumonia	0	(0%)	0	(0%)	NS
Sepsis	0	(0%)	0	(0%)	NS
Hospital mortality	9	(69%)	1	(13%)	0.03

*Median and interquartile range

## Discussion

Previously, the mortality of ATAAD with ACI was reported to be between 19.5 and 33% [2–4]. However, one of the most challenging aspects of ACI is a correct preoperative diagnosis and evaluation. Firstly, the main symptom of ATAAD “masks” that of coronary ischemia [5], and secondly, there is no definitive diagnostic examination.

As a diagnostic material for acute myocardial infarction, CK-MB is prevalently used. Since the etiology of the ACI due to ATAAD is different from that of ordinary coronary artery disesase, the ACI does not necessarily lead to myocardial necrosis at the same speed. However, it was unknown how many patients with ACI had a CK-MB elevation and the true impact of CK-MB elevation on the surgical outcome of ATAAD with ACI.

The present study showed that about 62% of patients with ACI had an increase in CK-MB. Moreover, the MI group had significantly higher mortality than the NMI group. As time is an essential factor when considering CK-MB, it should be noted that this study dealt with the data when surgery was performed about 4 h after the onset. If it took more time to operate, the proportion of patients with a CK-MB elevation would increase, and as a result, the mortality was also expected to be higher. Previous reports showed that time to operation was a predictive factor for patients with ATAAD [6, 7]. However, in patients with ACI, time management to prevent CK-MB from rising is important. PCI would be able to revascularize fastest, and there is a possibility that it can prevent CK-MB elevation.

Uchida et al. reported in detail the results of early reperfusion for the first PCI in 25 patients with ATAAD with ACI(8). Of the 25 patients, 14 had PCI at the beginning, followed by 11 with central repair, with a mortality rate of 0%. On the other hand, all patients who did not undergo central repair with/without PCI, including palliative care alone (*n* = 2), died. The overall mortality was 10/25 (40%). This study implied that central repair was essential for lifesaving. Considering that the MI group had a mortality rate of 69% and the NMI group had a mortality rate of 13%, the future task is to complete the treatment while performing central repair without increasing CK-MB as much as possible. Completing Aortic repair is necessary, and it is dangerous to pay attention only to precede PCI.

**Fig. 1:**
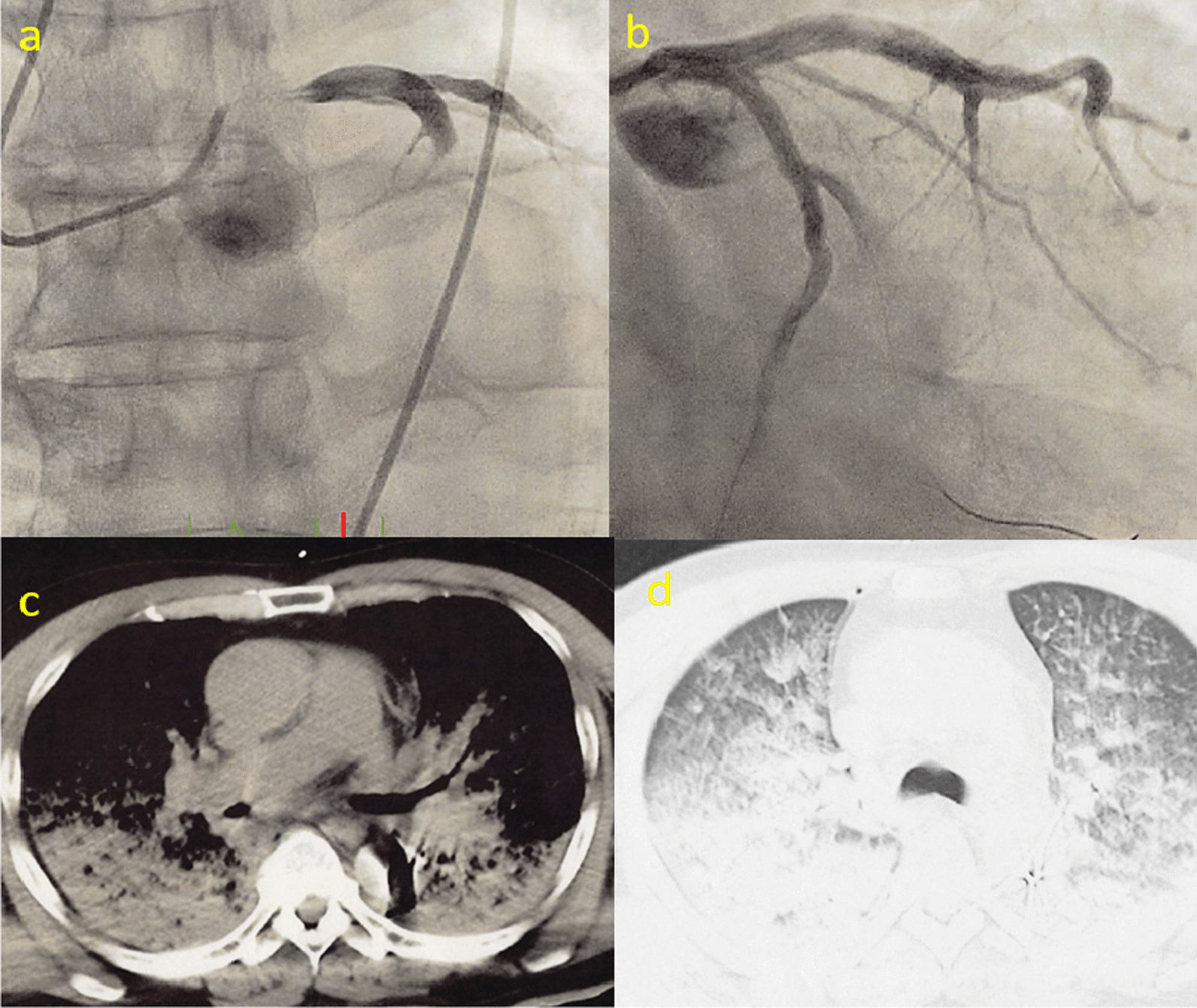
Severe ACI case with LMT obstruction. **a**: The LMT was dissected, and coronary flow was very slow. **b**: PCI for LMT was performed, but the flow remained slow. **c**,**d**: the patient suffered acute heart failure. IABP was seen in the descending aorta. LMT:, PCI:percutaneous coronary intervention, IABP: intra-aortic balloon pumping

Since it is not easy to diagnose ACI preoperatively, it would be difficult for cardiologists and cardiac surgeons to treat every emergency case practically. On the other hand, although it would take more time than PCI, additional CABG can be performed safely only by cardiac surgeons, which is more feasible. As for the timing of CABG, we performed CABG after aortic repair. We prioritized achieving cardiac arrest over revascularization of the coronary arteries because the progression of myocardial ischemia can be delayed by circulatory arrest. After all, it reduces myocardial oxygen demand by more than 90%. However, there is a situation where PCI first management is superior. In the most severe ACI cases, sudden occlusion of the coronary artery may result in death in tens of minutes. This study included only one case of such acute occlusion of the left main trunk (Fig. 1). Preoperative PCI played an important role as a bridge to operation in this case. Although the patient was in hemodynamically severe condition even after the PCI, he underwent the operation successfully. He survived five years of follow-up. In the real world, the degree of ACI, the transportation time to the surgical facility, and the medical level of the initial medical institution would vary for each patient. Therefore, it is a future research subject to clarify what kind of treatment is the best for each patient’s severity.

## Limitations

Our report described a retrospective and observational single-center non-randomized study. Secondary, the sample volume was small. However, ATAAD with ACI was rare, and the sample volume was not large even in past reports. Finally, we had no control group of patients with ACI not receiving CABG or undertaking PCI only without CABG. Further studies are required to clarify the best treatment.

## Conclusion

Sixty-two percent of patients with ACI increased CK-MB in an average of 4 h from onset to surgery. The mortality of patients with CK-MB elevation was significantly higher than those without CK-MB elevation. It is important to treat patients with ATAAD complicated with ACI before they have an increase in CK-MB.

## Data Availability

Data sharing not applicable to this article as no data sets were generated during the current study.

## Abbreviations

ACI: Acute coronary artery involvement
ATAAD: Acute type A aortic dissection
CK: Creatine kinase
CK-MB: Creatine kinase muscle and brain isoenzyme
PCI: Percutaneous coronary intervention
CABG: Coronary artery bypass grafting
RCA: Right coronary artery
LCA: Left coronary artery
